# Tools to Quantify and Characterize the Persistent Reservoir in People with HIV-1: Focus on Non-B Subtypes

**DOI:** 10.3390/v18010110

**Published:** 2026-01-14

**Authors:** Zora Sinay, Annefien Tiggeler, Robert-Jan Palstra, Tokameh Mahmoudi

**Affiliations:** 1 Department of Pathology, Erasmus University Medical Center, 3015 GD Rotterdam, The Netherlands; z.sinay@erasmusmc.nl (Z.S.); a.tiggeler@erasmusmc.nl (A.T.); r.palstra@erasmusmc.nl (R.-J.P.); 2 Department of Urology, Erasmus University Medical Center, 3015 GD Rotterdam, The Netherlands

**Keywords:** HIV-1, HIV-1 reservoir, reservoir quantitation, Non-B subtypes

## Abstract

Human immunodeficiency virus type 1 (HIV-1) continues to be a major global health burden. Combination antiretroviral therapy (cART) effectively abrogates HIV-1 replication and has transformed HIV-1 infection from a fatal to chronic disease. While ART can suppress viremia to undetectable levels in people living with HIV-1 (PWH), a small reservoir of cells infected with replication-competent HIV-1 persists and can lead to viral rebound upon ART interruption. This persistent HIV-1 reservoir can be quantified and characterized by measuring replication of infectious HIV-1 using a quantitative viral outgrowth assay (qVOA), or by measuring HIV-1 DNA, RNA, or protein levels as a proxy for the reservoir. Tools to quantify the reservoir in these distinct molecular compartments have been developed for HIV-1 subtype B, which is predominant in the Global North. However, non-B subtypes constitute the majority of HIV-1 infections worldwide. Here, we discuss the wide range of reservoir quantitation and characterization tools, explore their limitations, and, where applicable, their adaptations to non-B subtypes. We conclude that standardized tools should be used to characterize reservoir dynamics of HIV-1 B and non-B subtypes. These tests should be well-validated and accessible to all laboratories world-wide to be able to draw conclusions about subtype-specific reservoir dynamics.

## 1. Introduction

Human immunodeficiency virus (HIV) remains a major global health burden, with over 40 million people affected worldwide [1]. With the development of combination antiretroviral therapy (cART) in the mid-1990s, HIV-1 treatment was revolutionized, and HIV-1 infection changed from a lethal to a chronic condition [2,3,4,5]. Since the emergence of cART, treatment has become increasingly safer, easier to administer, and more effective [6,7,8,9]. Currently, a single daily ART tablet results in sustained viral suppression in nearly all individuals [7]. Consequently, the life expectancy of people living with HIV-1 (PWH) has been increasing and approaching that of HIV-negative populations [10]. Despite this success, chronic HIV-1 infection remains associated with various comorbidities and adverse effects to treatment [11,12,13,14,15]. HIV-1 infection is complicated by the long-term persistence of a small population of infected cells, collectively referred to as the persistent HIV-1 reservoir [16]. This reservoir is well known to drive viral rebound upon interruption of ART [17]. Consequently, the scientific community has devoted considerable effort to characterize and quantify these long-lived infected cells. A range of experimental quantitation and characterization tools have been developed to characterize the reservoir, each targeting distinct molecular intermediates of the HIV-1 life cycle, including viral DNA, RNA, and proteins (reviewed by Abdel-Mohsen et al. [18]). These tools are essential for elucidating the mechanisms that sustain reservoir persistence and assessing the effectiveness of cure strategies in clinical trials.

Thus far, reservoir quantitation tools have been largely developed to quantify the reservoir in people with HIV-1 subtype B, the predominant HIV-1 subtype in the Global North [19]. Most HIV-1 infections, however, are caused by non-B subtypes (88%) [19]. The term “non-B subtypes” refers to many genetically distinct subtypes (group M: A-D, F-H, and J-L) and recombinant forms. Of these subtypes, HIV-1 subtype C is the predominant subtype in India and Eastern and Southern Africa and accounts for roughly half of global HIV infections [19]. Regardless, the main body of HIV-1 pre-clinical studies are conducted in the Global North and have focused on subtype B characterization and treatment.

Due to the selective tool development for HIV-1 subtype B and the availability of cohorts and samples, the majority of the crucial studies on HIV-1 reservoir biology have been conducted exclusively on this subtype. It is therefore important to recognize that the current understanding of HIV-1 reservoir biology, including the timing of reservoir establishment, longitudinal dynamics, response to latency reversal agents (LRAs), and viral rebound after treatment interruption, cannot necessarily be generalized for non-B HIV-1 subtypes and remain incompletely understood for non-B subtypes.

To gain a more complete understanding of the HIV-1 reservoir in people with non-B HIV-1 subtypes, an increasing number of reservoir characterization and quantitation tools are now being adapted and developed specifically for these subtypes. In this review, we discuss these tools, their limitations, their predictive value, and their relevance for advancing the understanding of the HIV-1 reservoir of all PWH. To contextualize the importance of reservoir quantitation and characterization tools, we provide a global overview of the HIV-1 life cycle, cART, reservoir biology, and the role reservoir plays in the ongoing search for cART-free viral suppression.

### 1.1. HIV-1 Life Cycle

Throughout its life cycle, HIV-1 generates distinct macromolecular intermediates—such as genomic single-spliced RNA, proviral double-stranded DNA, various RNA transcripts, and fifteen viral proteins—that can all be studied using HIV-1 reservoir quantitation tools. The HIV-1 life cycle begins with viral attachment to host (co-)receptors and subsequent fusion of the viral membrane with the host cell to enable entry of the HIV-1 RNA genome into the cytoplasm [20]. The RNA genome is then reverse-transcribed into a double-stranded DNA (dsDNA) provirus, after which viral integrase catalyzes integration into the host genome [21,22,23,24]. Once integrated, the 5′ long terminal repeat (LTR) functions as the primary HIV-1 promoter and can initiate transcription [25]. After transcription, the full-length 9 kb viral RNA (vRNA) can be subjected to alternative splicing to generate various RNA species that are commonly classified as unspliced (usRNA), single-spliced (ssRNA) and multiply spliced RNA (msRNA) [26,27]. These vRNAs form ribonucleoprotein complexes that allow for trafficking of the RNA species out of the nucleus. In the cytoplasm, vRNAs can be translated into viral proteins that, combined with the genomic RNA, allow the budding of mature virions from the host [28,29].

### 1.2. Combination ART

cART effectively blocks viral replication by targeting distinct steps within the HIV-1 life cycle: fusion inhibitors and co-receptor antagonists prevent HIV-1 from entering the host cell; nucleoside/nucleotide inhibitors (NRTIs) and non-nucleoside reverse transcriptase inhibitors (NNRTIs) inhibit reverse transcription; integrase strand transfer inhibitors (INSTIs) prevent proviral integration into the host genome; and protease inhibitors inhibit proteolytic cleavage of the polyprotein Gag-Pol, blocking the maturation of the budded virions [6]. The necessity of combining multiple antiretroviral (ARV) drugs to achieve effective suppression of HIV-1 viremia can be attributed to the highly error-prone nature of the reverse transcription process, the second step in the HIV-1 replication cycle [30]. The success of cART can therefore be partly explained by its simultaneous targeting of multiple steps of the viral life cycle, which substantially lowers the likelihood of viral escape. Long-acting ART is an important recent advancement in HIV-1 treatment that relies on injectable ARV drugs to maintain effective viral suppression for extended periods, thereby eliminating the need for daily dosing [31,32]. The first long-acting formulation approved as an injectable ART regimen was monthly or bi-monthly injectable cabotegravir (integrase inhibitor) and rilpivirine (RT-inhibitor) in virologically suppressed PWH [33,34]. Additionally, lenacapavir, a long-acting capsid inhibitor, effectively maintains viral suppression with 6-monthly dosing in oral treatment-experienced individuals [35,36,37]. Through substantially reducing dosing frequency, LA-ART addresses key limitations of daily oral cART, including suboptimal adherence, treatment fatigue, and HIV-related stigma [38,39]. Important to note is that this new treatment avenue prompts novel questions regarding the impact of LA-ART on the longitudinal HIV-1 reservoir dynamics.

### 1.3. The Persistent HIV-1 Reservoir

Regardless of the success of cART, vRNA in plasma quickly rebounds if treatment is interrupted [17]. The biological driver behind this rebound viremia post-cART is the population of long-lived immune cells that harbor replication-competent provirus that constitute the persistent reservoir. The HIV-1 reservoir is established very early, within days of primary infection [40,41,42,43]. Evidence in non-human primates shows that very early initiation of cART could not prevent viral rebound after treatment-interruption [44]. Additionally, three separate studies in PWH also showed that while early cART initiation can reduce the reservoir size, its establishment cannot be prevented [45,46,47].

A crucial characteristic of the HIV-1 reservoir is that it consists of a heterogeneous population of cells that do not all contribute equally to viral rebound after cART cessation. For instance, over 90% of the persistent reservoir cells contain defective integrated proviral DNA and will not give rise to infectious virions upon cART interruption [45,48,49,50,51,52,53]. Studying intact versus defective reservoir dynamics revealed that cells harboring defective proviruses decay more slowly than those with intact proviruses, indicating that defective proviruses either escape immune clearance more effectively or undergo a different balance of cellular turnover compared to intact proviruses [54,55,56]. Additionally, defective proviruses have been shown to express aberrant proteins, and cells that express these defective proteins can mask the actual replication-competent cells, preventing their clearance by cytotoxic T cells [57]. These important insights are co-facilitated by the ability to quantify the total, intact, and defective proviral DNA reservoir, highlighting the relevance of DNA-based reservoir quantitation tools.

As the HIV-1 reservoir complexity extends beyond the intactness of integrated provirus, reservoir quantitation tools have also been developed to target transcriptional and post-transcriptional intermediates of HIV-1. Essential context for this review is the ongoing debate in the field of HIV-1 research on the transcriptional state associated with HIV-1 [58]. Traditionally, the HIV-1 reservoir has been referred to as the latent reservoir, where ‘latency’ refers to transcriptional silence. Recently, this traditional view of reservoir cells as HIV-1 DNA-positive but HIV-1 protein- and RNA-negative has been challenged by multiple studies showing the presence of a population of reservoir cells that is transcriptionally “leaky” while not producing virions on cART [40,41,42]. Co- and post-transcriptional blocks are suggested to inhibit these transcriptionally active reservoir cells, preventing the production of virions [59]. We therefore use the term “persistent reservoir” instead of “latent reservoir” to acknowledge the transcriptional activity of reservoir cells.

An important question—that also has been addressed through thorough reservoir quantitation and characterization—is how the reservoir of HIV-1 subtype B is remarkably stable over time. Once seeded, the proviral reservoir in CD4+ T cells decays slowly, with a reported half-life of multiple decades [54,56,60,61,62,63]. While the size of the persistent HIV-1 reservoir is therefore stable, it is not static and is marked by dynamic changes over time [64,65]. The decay of the reservoir plateaus after 4 to 7 years on suppressive cART [63]. The mechanism behind this long-term persistence and decay is thought to be the changing balance between reservoir size increase by clonal expansion and decay by immune clearance through adaptive killing by cytotoxic T cells and natural killer cells [66,67]. Clonal expansion can be driven by specific antigen recognition but can also be homeostatic to maintain the repertoire of memory CD4+ T cells [68,69]. Interestingly, the proliferation of reservoir cells has also been shown to be integration site-dependent, as the retrovirus can integrate into genes that promote cellular survival, leading to a growth advantage and expansion of that clone (reviewed by Yeh et al. [64] and Liu et al. [70]). Interestingly, over 50% of intact proviruses originate from expanded clones, which suggests that the absolute number of initial reservoir cells that are seeded is greatly supplemented by clonal expansion mechanisms [54,71]. These insights are fundamental to developing curative approaches that aim to silence, control, or eliminate reservoir cells and have all been enabled by the development of reservoir quantitation and characterization assays.

Moreover, a crucial recent insight into the HIV-1 subtype B reservoir is that not all integrated proviruses are capable of reactivation due to the location of genomic integration in heterochromatin, characterized by tightly packed DNA that is transcriptionally silent, constituting a repressive chromatin region [52,72,73]. Interestingly, in long-term cART-suppressed individuals, the integration of the provirus shifts to predominantly heterochromatic regions over time [42,52,53], an observation that can be explained by the earlier mentioned balance between clonal expansion and immune clearance. While immune clearance selects against proviruses that are present in permissive chromatin and hence (partly) transcriptionally active, proviral integrations into heterochromatin may accumulate through clonal expansion [52,53]. This results in a reservoir that, gradually, becomes increasingly transcriptionally silent through enrichment of cells that are “locked” in repressive chromatin structures.

All in all, the HIV-1 subtype B reservoir and its dynamics are increasingly well-characterized, leading to a deeper understanding of the mechanisms of its persistence, reactivation, and repression, which is critical for the development of targeted cure strategies.

### 1.4. Genetic Diversity and HIV-1 Subtypes

As mentioned, reservoir dynamics have mostly been described for HIV-1 subtype B, despite the fact that the majority of HIV-1 infections are caused by non-B subtypes [19]. HIV-1 emerged through multiple cross-species transmissions of simian immunodeficiency virus (SIV) from non-human primates to humans [16]. These distinct transmission events gave rise to separate HIV-1 groups: major (M), outlier (O), N, and P. Group M viruses have dominated the global pandemic, and this group can be further subdivided into ten genetically distinct subtypes (A–D, F–H, and J–L). In addition, HIV-1 can undergo recombination in individuals co-infected with multiple HIV-1 strains. These recombinant forms are referred to as circulating recombinant forms (CRFs). Currently, 169 CRFs have been deposited in the Los Alamos National Laboratory (LANL) HIV sequence database [74].

**Figure 1:**
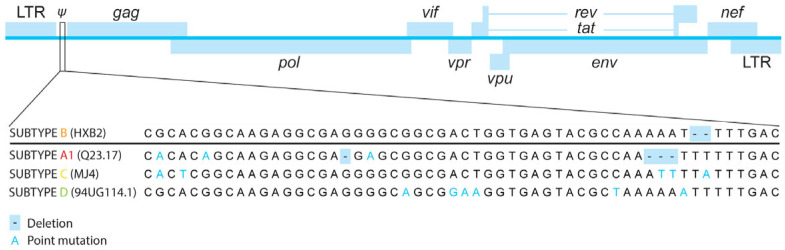
**Subtype-specific nucleotide differences between most prevalent HIV-1 subtypes.** Representative sequence alignment showing nucleotide differences between HIV-1 subtype B and most prevalent non-B subtype sequences in *psi* (HXB2 714-766), a common target in quantitative DNA assays. The nucleotide sequences are based on published HIV-1 sequences from clinical isolates and compared to HXB2 to show inter-subtype differences. Deletions are written as “-” and marked in blue, and point mutations compared to the subtype B sequence are shown as blue nucleotide bases (see legend).

While the structure of the HIV-1 genome is conserved amongst subtypes, proviruses show genetic inter-subtype variation in nucleotide sequences and, therefore, amino acid sequences (Figure 1). Functional differences caused by inter-subtype variation can affect both acute HIV-1 infection and reservoir dynamics. For instance, Rev of HIV-1 subtype C is typically 16 amino acids shorter than Rev of other subtypes [75]. While a C-terminal truncation does not appear to affect Rev activity, it does regulate Rev stability [76]. When discussing the HIV-1 reservoir, the 5′ LTR is of particular interest as it contains the HIV-1 promoter and multiple transcription factor binding sites, such as NF-κB and Sp1 motifs [77]. The concerted activity of the activating and repressive transcription factors and their co-factors regulates the activity of the HIV-1 promoter. Genetic variation between subtypes has led to differences in transcription factor binding sites that have major effects on transcription and its regulation. This is seen in the 5′ LTR of HIV-1 subtype C, which contains at least one extra NF-κB enhancer motif compared to the 5′ LTR of subtype B [78]. While this may indicate increased susceptibility to stimulation, multiple studies have shown that subtype C has a weaker response to agents that stimulate through NF-κB activation than subtype B [79,80]. In addition, the extra NF-κB motif appears to have a stimulatory effect on immune evasion. HIV-1 subtypes A, B, and D, which only have two NF-κB motifs in their LTR, can be inhibited by interferon-induced protein IFI16, which sequesters Sp1, because their transcription is partly dependent on Sp1 [81]. Conversely, a third NF-κB motif, as seen in subtype C, has been associated with resistance to IFI16 inhibition due to a lack of dependency on Sp1 for transcription [82]. These findings underline the substantial differences in behavior between HIV-1 subtypes as a result of genetic diversity and the need for research into these functional differences.

Although few studies have examined non-B reservoirs, noteworthy findings have been documented [83]. Two separate studies compared the reservoir size between an American subtype B cohort and a Ugandan cohort with infections of subtype A1, C, and D [84,85,86,87]. Both studies found that subtype B reservoirs were larger than any of the non-B reservoirs. These findings were substantiated by other studies that compared reservoir size between non-B and B subtypes and found that non-B reservoirs (A-H and CRFs) tend to be smaller in size than subtype B reservoirs [84,85,86,87]. Interestingly, Bachmann et al. found a trend of non-B reservoirs decaying faster than subtype B reservoirs [84]. It is unknown whether increased decay accounts completely for the observed smaller reservoir sizes. Additionally, Tso et al. found a considerably smaller tissue reservoir in the brain in people with HIV-1 subtype C than those with HIV-1 subtype B [88]. Furthermore, non-B reservoirs have been found to be genetically more diverse when compared to subtype B [89]. Similarly, a large genetic diversity in the reservoir was reported in a study where viral sequence evolution in people with HIV-1 subtype C was assessed before and after treatment [90]. Finally, Reddy et al. showed that hyperacute diagnosis of women with HIV-1 subtype C did not impact reservoir seeding but was associated with a rapid decay of intact viral genomes and decreased genetic complexity [45].

When assessing what is known about the reservoir dynamics of non-B subtypes, it is important to take into account how understudied subtype-specific reservoir dynamics are. While the aforementioned studies have found significant differences, many studies were conducted in specific regions and sometimes with few study participants per subtype. These limitations to the currently available data highlight the urgence of studying each non-B subtype independently for subtype-specific differences in reservoir dynamics.

### 1.5. Strategies to Silence, Control, or Reduce the Reservoir

Most efforts to quantify and characterize the HIV-1 reservoir are driven by the search for a curative intervention for HIV-1. A cure for HIV-1 is defined here as controlled viremia in the absence of ART, and there are three general strategies described to achieve a cure: (1) silencing, (2) controlling, and (3) reducing the HIV-1 reservoir. For the first cure strategy, to silence the reservoir, the so-called “block-and-lock” approach is focused on permanently inactivating HIV-positive cells to eliminate all viral transcription and inducing resistance to reactivation in these cells [91]. This can be established through epigenetic silencing by RNA-based mechanisms. Short interfering RNA (siRNA), short hairpin RNA (shRNA), and other RNA transcripts can recruit chromatin-remodeling complexes that induce repressive epigenetic changes, thereby silencing vRNA expression [91]. Another strategy that is explored to silence the HIV-1 reservoir is inhibiting the viral protein Tat, which is essential for reactivating latently infected cells. Consequently, targeting and inhibiting Tat represents a viable approach to selectively suppress the HIV-1 reservoir. Tat inhibition can be modulated by small molecule inhibitors (SMIs) that either target Tat itself [92,93] or Tat interactors [94,95]. In addition, SMIs can be used to steer integration towards heterochromatin to render them transcriptionally silent [96], or to target signaling pathways involved in the reactivation of latent HIV-1, such as the mTor signaling pathway [97]. Finally, spironolactone, a cardiovascular drug, has been shown to temporarily silence (viral) transcription and reduce immune activation [98,99]. The overall aim of the “block-and-lock” strategy is to eliminate all viral transcription, irrespective of whether transcription stems from intact or defective DNA. Therefore, quantitation of cell-associated viral RNA transcripts would be appropriate to assess suppression of viral transcription via candidate “block-and-lock” interventions.

The second strategy toward an HIV-1 cure is to control viral rebound after ART interruption. To this end, ex vivo genome editing by CRISPR/Cas9 is an interesting approach explored to generate CCR5/CXCR4 co-receptor-deficient hematopoietic stem cells [100]. The objective for this approach is to replenish the immune system of PWH with immune cells that are resistant to HIV-1. While tissue-resident HIV-positive cells may not be fully eliminated during this procedure, reactivation of residual HIV-positive cells would not lead to further infection of HIV-negative cells, as they will be resistant to HIV-1 infection. The success of the CRISPR/Cas9 genome editing can easily be assessed through sequencing of the target site, as well as by measuring CCR5 or CXCR4 expression with flow cytometry. Another approach to control viremia is to utilize the immune system to eliminate either cell-free virus or HIV-positive cells. For instance, broadly neutralizing antibodies (bNAbs) that target conserved regions in the envelope protein Env can be used to intercept cell-free virus to suppress viremia [101]. The association of bNAbs with Env prevents the attachment of virions to CD4 and subsequent entry into cells, which again would halt the spread of HIV-1 to uninfected cells after reactivation. In addition, bNAbs contain an Fc-domain that enables the elimination of cell-free virions, as well as the cytotoxic killing of HIV-positive cells. Moreover, many different therapeutic vaccines have been developed to increase the cytotoxic immune response against HIV-positive cells [102]. Different targets have been used for immunization with therapeutic vaccines, including Env, Gag, Pol, and Nef. The effectiveness of approaches that rely on immune clearance of HIV-positive cells can be evaluated by longitudinal quantitation of the frequency of translationally competent reservoir cells.

The aforementioned techniques to control viremia have also been explored as a means to reduce the reservoir after intentional reactivation of the reservoir. A large body of this research has focused on the so-called “shock-and-kill” approach, where persistent reservoir cells are “shocked” with LRAs to induce expression of the viral RNA and protein, rendering cells susceptible to either undergo induced cell death (ICD) or to produce immunogenic antigens that target reservoir cells for immune clearance [103]. The immune system of PWH is largely dysfunctional; however, it can be strengthened via several immunomodulatory strategies, including therapeutic vaccination [104,105], adoptive cell transfer of CAR-CD8^+^ T cells [106], immune checkpoint blockade [107], bNAbs [108], and other immunotherapeutic molecules that engage T and NK cell cytotoxicity to eliminate reservoir cells [108]. Aside from harnessing the immune system, ICD of the reservoir cells can also be induced by pharmacologically triggering innate intracellular pathways that induce apoptosis selectively in LRA-reactivated cells [109]. Examples of ICD-inducing compounds are SMAC mimetics [110], TACK molecules [111], and DDX3 inhibitors [112], each of which relies on a distinct molecular mechanism to induce apoptosis. Important to note is that LRA-induced reactivation of the reservoir should induce viral transcription and protein expression by removing transcriptional and post-translational blocks [41]. To evaluate the efficacy of “shock-and-kill”-based reservoir size reduction, both (inducible) vRNA as well as (intact) DNA levels should be assessed. Another avenue explored to reduce the reservoir involves CRISPR/Cas9 genome editing to directly inactivate or remove the HIV-1 provirus from the genome of host cells [100,113]. To assess the effectiveness of these genome editing approaches, specific proviral DNA regions affected by the genome-editing intervention should be interrogated and quantified using specific primers. Finally, viral outgrowth assays can indicate whether the gene therapy approaches successfully reduce replication-competent reservoir.

## 2. Main

HIV-1 proviruses are hallmarked by genetic diversity, both between subtypes and within the same subtype. This genetic diversity of HIV-1 necessitates a shift in research to more relevant non-B subtypes in populations that are most affected by HIV-1. The development of tools that quantify and characterize all circulating HIV-1 subtypes worldwide is essential to close the current gap in HIV-1 research. In the next section, we provide an overview of the wide scope of reservoir quantitation tools that have been developed to assess molecular intermediates of various stages of the viral life cycle (DNA, RNA, protein, and virion). We conclude the review by discussing the assays that have already been used for the quantitation and characterization of non-B reservoirs, obstacles to adaptations of other methods, and possible ways to overcome these limitations.

### 2.1. Viral Outgrowth Assays

After ART interruption, reservoir cells harboring intact replication-competent proviruses can initiate new rounds of replication after reactivation. The golden standard for quantifying the replication-competent reservoir size is the quantitative viral outgrowth assay (qVOA). In a qVOA, PBMCs (peripheral blood mononuclear cells) or (resting) CD4+ T cells from PWH are cultured with uninfected cell lines or PBMCs from a healthy donor (HD), and the supernatant is assessed to quantify released virions. Levels of p24 or vRNA in the supernatant are used to determine how many cells in the culture produce infectious HIV-1. In this chapter, the different types of qVOAs will be discussed, as well as their limitations and alternative assays to obtain virus or study virus production.

#### 2.1.1. Conventional qVOAs

In the first qVOA protocol, dilutions of PBMCs from PWH were co-cultured with IL-2 and activated PBMCs from HDs for 28 days [114]. Concentrations of p24 were then measured to determine the lowest number of PBMCs required to produce positive cultures. PBMCs from virally suppressed PWH, however, contain few cells that can produce infectious virions, requiring large cell numbers to successfully perform qVOAs [115]. Due to the tropism of HIV-1 for CD4+ T cells [116], later qVOAs are performed using (resting) CD4+ T cells of PWH [117,118,119,120]. Results from qVOAs have since been expressed as infectious units per million (IUPM) CD4+ T cells. A second change to the original qVOA protocol was the addition of stimulation of the CD4+ T cells before or during co-culture with potent stimulants like PHA, PMA and ionomycin, anti-CD3 and anti-CD28, and/or irradiated HIV-1 PBMCs to induce virus production [117,119,120,121,122,123]. While many variations in the qVOA are used to quantify the HIV-1 reservoir, all assays report a measurement of the frequency of infectious HIV-positive CD4+ T cells.

##### Limitations

Since the qVOA is one of few assays that assesses virus production and measures the number of cells containing the infectious HIV-1 genome, it is understandable that the qVOA is generally regarded as the golden standard. However, the predictive value of the qVOA for viral rebound after ART cessation remains debated. Sequencing studies have shown poor correlation between virions produced during the qVOA and virions produced during viral rebound [17,124,125,126,127]. This indicates that the virions produced during viral rebound may not originate from CD4+ T cells in the blood but rather from reservoir cells in other cellular compartments. A qVOA on cells isolated from blood may therefore hold limited predictive value for viral rebound.

Furthermore, the qVOA leads to an underestimation of the HIV-1 reservoir. Ho et al. have shown that stimulation with PHA does not lead to the induction of all replication-competent proviruses [50]. Beliakova-Bethell et al. showed that indeed anti-CD3/anti-CD28 are more efficient in inducing the transcription of replication-competent proviruses after 14 days than PHA or PMA with ionomycin [128]. In addition, not all reactivated cells produce enough virus to initiate viral outgrowth—leading to false-negatives [129]. It is therefore likely that reservoir quantitation by qVOA underestimates the true size of the reservoir. The reliability of the qVOA data is further queried by the finding that the qVOA shows great inter- and intra-laboratory variability [130].

A final limitation of the qVOA is that the protocols are generally considered to be costly and laborious. One costly element of the qVOA protocol is the requirement for PBMCs from HDs. In response, multiple qVOAs have been developed that utilize cell lines instead of HD PBMCs [121,122,131,132]. These cell lines can be kept in culture continuously, and therefore severely reduce the burden of the protocol. A comparative study showed that qVOAs with cell lines and qVOAs with HD PBMCs correlate strongly when viral protein is measured [133]. A second factor contributing to the burden of the qVOA is the duration of the protocol. Conventional qVOAs take 14 to 28 days to execute [114,118,119,120,134]. To address this, multiple protocols have been developed that require 7 to 12 days of culturing [117,132,135]. These limitations of the qVOA protocols restrict its application for fast high-throughput screening of the reservoir.

#### 2.1.2. Alternative Assays

The qVOA does not always lead to virus release from reservoir cells [50,136]. In particular, for samples from elite controllers, PWH that efficiently suppress viremia without ART, it is difficult to induce virus production. Metcalf Pate et al. designed a qualitative assay to recover virus from samples that are hard to induce in qVOAs, the murine viral outgrowth assay (mVOA) [137]. In this assay, leukocytes from PWH are transferred into CD8+ T cell-depleted humanized mice. The rationale is that this transfer of infectious tissue will trigger viral expression, and virus can be obtained from cells from virally suppressed PWH. This was confirmed with samples from five PWH and six elite controllers. Since then, more mVOAs have been developed, as reviewed by Schmitt and Akkina [138]. The sensitivity of these assays allows for virus recovery in samples that fail in qVOAs. Schmitt and Akkina argue that mVOAs could therefore play an important role in assessing the effectiveness of LRAs.

Another variation in the qVOA is the virus release assay (VRA). In this assay, PBMCs or resting CD4+ T cells are cultured with and without stimulation [139]. After 7 days of culturing, RNA in virions in the supernatant is measured. Cillo et al. showed that HIV-1 DNA and cell-associated vRNA levels in suppressed PWH correlated with the quantity of released virions during the VRA [139].

### 2.2. DNA Assays

The genome of retroviruses such as HIV-1 is integrated into the host genome. Once integrated, the provirus remains there indefinitely. Theoretically, any provirus could trigger viremia post-ART. In this section, we will discuss the different assays that have been developed to quantify HIV-1 DNA and their relevance in characterizing the HIV-1 reservoir.

#### 2.2.1. PCR Assays

An infected cell contains one integrated copy of the HIV-1 genome [140]. One copy spans over 9 kb, a relatively small number of base pairs compared to the vast size of the human genome. To detect the provirus, it is therefore necessary to amplify the HIV-1 DNA. The polymerase chain reaction (PCR), a fundamental assay for the amplification of DNA, requires primers that flank the DNA target. A PCR is most efficient if the primers are very specific for the DNA of interest and if the amplicon is limited in length. For HIV-1, the first PCR, developed by Kwok et al. [141], targeted a small part of the *gag* gene, and the resulting amplified product was analyzed and quantified by gel electrophoresis. Primer sets have since been developed to amplify different regions within the HIV-1 genome [142].

The quantitative PCR (qPCR), an important improvement to the PCR, allows for quantitation of the DNA of interest during amplification. qPCR assays usually utilize a dye that fluoresces when it binds to dsDNA and, therefore, gives an increase in signal when DNA is amplified. Another way to perform a qPCR is to measure the amplification of the gene of interest with a probe, a single-stranded nucleotide sequence with an attached fluorophore. Currently, a common read-out for the HIV-1 reservoir is “total HIV-1 DNA”, which is usually determined with a qPCR of viral *gag* or *pol* genes.

##### Multiplexed PCR Assays

**Figure 2:**
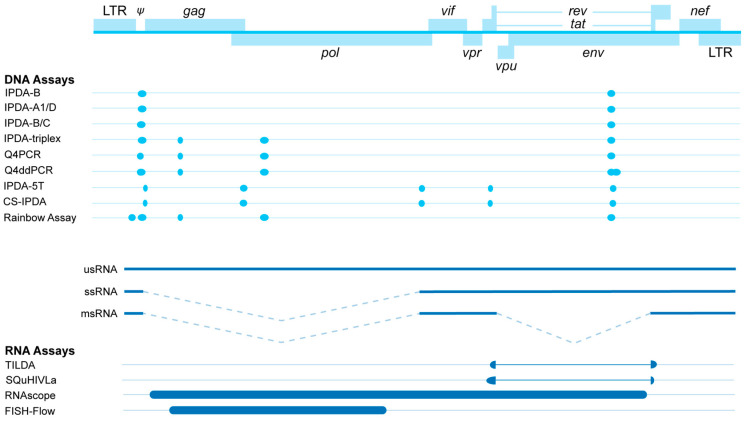
**Amplicon/probe positions in HIV-1 nucleotide-based reservoir quantitation techniques.** Schematic showing the distinct amplicon/probe positions (blue dots) in the nucleotide-based assays discussed in this review. Multiplexed DNA reservoir quantitation techniques amplify different sites to determine the intactness of HIV-1 DNA [144,145,146,147,148,149,150,151,152]; inducible msRNA assays TILDA and SQuHIVLa use primer sets that span the *tat*/*rev* intron [153,154]; and in situ hybridization techniques RNAscope and FISH-Flow use, respectively, 78 and 50 target probes along a large part of the HIV-1 genome [155,156]. usRNA, unspliced RNA; ssRNA, single-spliced RNA; msRNA, multiply spliced RNA.

While total HIV-1 DNA is often measured in clinical trials, it is known that the majority of integrated HIV-1 proviruses are defective, with only a small proportion of the integrated proviruses being intact [48,49,50,143]. In addition, the proportion of cells that contain replication-competent proviruses as determined by a qVOA is even lower [49,50]. These findings have led to the distinction between quantifying total HIV-1 DNA and intact HIV-1 DNA. Recently, many assays have been developed to measure intact HIV-1 DNA to quantify a more relevant reservoir. These assays rely on multiplexing probe-based qPCRs. Multiple regions in the HIV-1 genome are amplified (Figure 2), and probes with different fluorophores are used to measure the signal from each region.

The intact proviral DNA assay (IPDA) is a multiplexed probe-based digital droplet PCR (ddPCR) that targets two well-conserved regions in *gag* and *env* across the HIV-1 genome that are often deleted or otherwise mutated in defective proviruses [144]. In the IPDA, an intact provirus is defined as a double positive signal. Interestingly, proviruses with deletions or hypermutations in *env* are far more common than proviruses with deletions in *gag*. The IPDA correlates to the qVOA with a Pearson’s r value between 0.48 and 0.78 and reports anywhere between a 10- to 1000-fold increase in absolute values [133,144]. While the qVOA usually results in an underestimation of the replication-competent reservoir, the IPDA demonstrates an overestimation.

In parallel, another probe-based qPCR assay was developed: the Q4PCR [148]. In this assay, a limiting dilution of CD4+ T cell DNA isolated from PWH is used to quantitatively assess the frequency of provirus through a *gag* PCR. A near-full-length (nFL) amplification of the provirus is then performed. This amplified product is diluted to single copies based on the previous *gag* PCR and used to quantitatively measure *psi*, *gag*, *pol*, and *env*. Any combination of at least two signals is considered intact. This method, too, was compared to a conventional qVOA and correlated with a Pearson’s r value of 0.63. The authors argue that the increase in targets compared to the IPDA makes the Q4PCR a more sensitive method, as potential polymorphisms are less likely to affect the assay. A downside to this technique is that the nFL sequencing step is inefficient, and therefore the method may actually lead to an underestimation of the intact HIV-1 reservoir [157]. Recently, this method has been adapted to reduce the labor involved with Q4PCR [149]. This Q4ddPCR is performed with a ddPCR that allows for the amplification and detection of all four regions of the original Q4PCR. This method eliminates the need for a limiting dilution *gag* PCR and separate nFL amplification. The reservoirs reported by the newer Q4ddPCR correlate with the reservoir as measured by Q4PCR (Spearman’s r = 0.92) and IPDA (Spearman’s r = 0.61–0.72). The authors also showed that the specificity of a multiplexed DNA assay increases with increasingly more targets, but that the sensitivity is reduced with every additional target. As a result, the Q4ddPCR reports ~40% fewer intact proviruses than the IPDA.

Levy et al. published a third ddPCR assay, the IPDA-5T [150]. This assay consists of five primer/probe pairs targeting LTR/*gag*, 5′ *pol*, 3′ *pol*, *tat*, and *env*. The IPDA-5T can be performed on a two-channel ddPCR machine because the targets are split over two different assays. This method showed poor correlation to the qVOA (Pearson’s r = 0.36 and Spearman’s ρ = 0.48).

Then, a fourth digital PCR (dPCR) assay, the triplex assay, targets three different sites in *psi*, *env*, and a third target, either *gag* or *pol* [147]. Both the *gag* and *pol* triplex assays detect a smaller number of intact proviruses than the IPDA and are equally reproducible. The triplex assays, like the Q4PCR [148], are more sensitive than the IPDA, but eliminate the labor that comes with the limiting dilution of the Q4PCR [147].

Finally, Delporte et al. recently published the Rainbow assay [152]. This dPCR targets five regions in the HIV-1 genome: LTR, *psi*, *gag*, *env*, and *pol*. The authors reported that the assay was less affected by polymorphisms than the original IPDA, which only amplifies two sites. Since the Rainbow assay probes five sites instead of two, fewer proviruses are identified as intact, leading to less overestimation of the reservoir.

##### Limitations

The multiplex DNA assays only probe between one and five regions of the HIV-1 genome, still allowing mutations in un-probed regions to go unnoticed. In addition, not all intact proviruses are replication-competent. As Einkauf et al. showed, transcriptional activity of the HIV-1 genome is dependent on activating histone acetylations and methylations [42]. This is in line with the finding that intact proviruses are mostly located in heterochromatin in elite controllers [51]. Therefore, PCR-based assays tend to overestimate the replication-competent reservoir size.

Furthermore, all PCR-based assays that quantify the intact HIV-1 reservoir depend on the amplification of specific regions of the HIV-1 genome. Comparative studies have shown that 6.3–28% of IPDAs fail due to polymorphisms preventing proper amplification and/or detection [158,159]. A major focus in research has been to increase the sensitivity and eliminate the effects of polymorphisms in these assays. One approach is to increase the number of probed regions, which is, for instance, applied in the Q4PCR assay. Comparative studies report that IPDAs generally result in a 19- to 42-fold higher reservoir measurement than the Q4PCR [157,160]. Another approach is to use donor- or cohort-specific primer and probe sequences, as showcased by Gunst et al. [108].

Lastly, several multiplexed DNA assays are run on a ddPCR machine that divides sample into droplets. This process can be laborious and time-intensive. Tumpach et al. adapted the IPDA as designed by Bruner et al. for a dPCR machine to eliminate the limitations associated with the ddPCR machine [161]. Results of the adapted IPDA were strongly correlated to the results with the original IPDA (Spearman’s r = 0.93), showing that the chip-based IPDA is a viable alternative to the original IPDA and also more accessible. Recently, Tschumi et al. described factors to consider in adapting a droplet-based IPDA to a chip-based IPDA [162]. They found that in 29.6% of clinical samples of subtype B, intact proviruses were not detectable, pointing to a potential need to run quadruplicates instead of duplicates to increase the chances of detection.

#### 2.2.2. Quantitative Integrated DNA Assays

HIV-1 is an RNA virus that is reverse-transcribed to DNA and subsequently integrated into the host genome, where it remains indefinitely. Not all proviral DNA present in cells is integrated. Unintegrated DNA is mostly transcriptionally silent, and it is degraded over time in dividing cells (as reviewed by Goff [163]). Hence, it is particularly interesting to measure the level of integrated DNA and the effect of any treatment on this reservoir.

There are multiple ways to measure integrated DNA levels. Most commonly, integrated DNA is measured through a nested *Alu*-LTR/*gag* PCR [164,165,166]. A nested PCR uses two subsequent PCRs with two different primer sets to increase sensitivity of detection. An *Alu* element is a short repetitive DNA sequence that is highly abundant in the human genome. The vast majority of the proviruses integrates close to an *Alu* element, with a distance of less than one nucleosome [167]. In an *Alu*-LTR/*gag* PCR, one primer is specific for *Alu* and the other primer is specific for the LTR or *gag* of the provirus. This ensures that only proviral DNA that is near an *Alu* element, and thus is integrated, is amplified. Earlier *Alu*-LTR/*gag* PCRs used Southern blots to quantify the integrated DNA, but more recent studies use probe-based qPCRs for quantitation [168,169,170]. A comparative study showed that measurements of integrated DNA correlate to IUPM as determined by qVOA (Pearson’s r = 0.70) and to levels of total HIV-1 DNA (Pearson’s r = 0.63) [171].

Another method for measuring integrated HIV-1 DNA is the inverse PCR [172,173]. This method consists of digesting only genomic DNA with an enzyme that cuts in the host and proviral DNA. Next, DNA is ligated to form circular DNA that spans from the restriction site in the provirus to the closest upstream restriction site. The DNA is then quantified by Southern blot. Similarly, Vandegraaf et al. published an alternative linker-PCR assay [174]. In this assay, DNA is digested, and linkers are added to the digested ends, which allows primers to bind for amplification. The product is subsequently measured by Southern blot. Finally, integrated HIV-1 DNA can also be measured by isolating high-molecular weight DNA from a gel and measuring HIV-1 DNA levels by qPCR [175,176].

#### 2.2.3. Proviral DNA Sequencing Assays

Next to quantitative assays, there are sequencing approaches that provide characterization of HIV-1 DNA reservoir dynamics. In 2005, Palmer et al. published single-genome sequencing (SGS) that allowed for the detection of rare mutations [177]. In this method, a nested PCR is performed on a single copy of the HIV-1 genome in plasma, which can then be sequenced by Sanger sequencing. Other methods allow for the sequencing of a single proviral copy [140,178]. Together, these methods enable the assessment of evolution of the HIV-1 provirus within one person or even one cell population.

A limitation of these techniques is that only part of the HIV-1 genome is amplified and sequenced. This prevents full understanding of the evolution and intactness of the HIV-1 genome. To address this, many techniques have been developed that allow for nFL amplification and sequencing of the HIV-1 provirus (as reviewed by Moar et al. [179]). Hiener et al. developed the full-length individual proviral sequencing (FLIPS) assay to amplify and sequence nFL HIV-1 proviral genomes [48]. In the FLIPS assay, two nested PCRs are performed to amplify full-length proviruses followed by next-generation sequencing (NGS). This technique was used to corroborate earlier findings that a large part of the integrated HIV-1 DNA is not intact [48,49,50]. The FLIPS assay has been combined with an in situ RNA hybridization method, FISH-Flow, to allow for sequencing specifically of reactivated vRNA+ reservoir cells [180]. This assay revealed that reactivated cells mostly contain defective proviruses. Sannier et al. also showed that identical clones reactivate differently, indicating varying transcriptional and (post-)translational blocks to expression.

nFL sequencing has been paired in multiple workflows with integration-site analysis to obtain information about the integration of intact and defective DNA [52,181,182,183]. One such method, matched integration site and proviral sequencing (MIP-seq), uses whole genome amplification (WGA) to amplify all DNA [52]. The amplified product is then split for integration-site analysis and nFL sequencing. MIP-seq has been used to show that intact DNA mostly integrates in non-genic DNA.

Finally, there are a few other applications for sequencing in HIV-1 research. Wu et al. used ASAP-seq, a single-cell ATAC sequencing method that is combined with immunophenotyping, to correlate the epigenetic landscape to the expression of cell surface markers [184]. Similarly, Sun et al. and Delley et al. paired proviral sequencing with phenotypic profiling in PheP-Seq and DAb-Seq, respectively [185,186]. These methods use barcoded antibodies to phenotype cells and to subsequently connect the phenotypic profiles to HIV-1 sequences from the same cells. Comparative analysis of the data showed that, in the comparison between HIV-positive vs. HIV-negative cells, one method showed significantly decreased expression of CD25, CD31, CD137, and CD278, while the other method showed no significant changes in expression [186].

##### Limitations

The majority of sequencing techniques that have been developed for HIV-1 depend on limiting-dilution approaches to amplify single-genomes in a PCR. The dilution of DNA to single genomes, followed by PCR and processing of every single well, is labor-intensive, time-consuming, and costly. To this end, the HIV proviral UMI-mediated long-read sequencing (HIV-PULSE) was developed [187]. In this method, HIV-1 proviruses are marked with unique molecular identifiers (UMIs), allowing PCR to be performed in bulk. The development of this easier and cheaper method enables high-throughput sequencing. Another solution to the high costs of most sequencing methods is nanopore sequencing. Cost-effective nanopore sequencing has been developed for multiple HIV-1 subtypes, including CRFs [188,189].

A second major limitation to the nFL sequencing techniques is that the long-distance PCR prior to sequencing is error-prone [190]. These methods fail to amplify roughly 70% of full-length sequences, biasing sequencing efforts against intact HIV-1 DNA. Quantitative data from sequencing is therefore unreliable in the distinction between intact and defective DNA.

#### 2.2.4. Integration Site Analysis

A specific application of sequencing efforts is the integration site analysis. Since transcription is partially dependent on chromosomal structure, the integration site of proviral DNA may have a significant influence on the reactivation of proviral DNA. There are multiple methods to determine integration sites. The HIV-1 integration site loop amplification (ISLA) assay uses specific primers to form loops of 3′ LTR HIV-1 DNA and adjacent chromosomal host DNA [191]. These DNA loops are then amplified and sequenced. Similarly, another method relies on the digestion of chromosomal DNA and subsequent ligation through linker-DNA to form loops that contain integrated HIV-1 DNA [192]. The loops are amplified using primers that bind to both the linker sequence and the 3′ LTR of the HIV-1 proviruses, and PCR products are then cloned into plasmids that are then sequenced. Finally, Sunshine et al. developed a probe-based method to determine integration sites [193]. In this method, genomic DNA is sheared by sonication and amplified, and subsequently, a pull-down is performed with probes to enrich for HIV-1 DNA. The DNA fragments are then amplified again and sequenced.

With the integration site analysis, the chromosomal landscape of the provirus can at least partially be determined. Interestingly, Einkauf et al. combined sequencing with the corresponding analysis of integration sites and vRNA expression in PRIP-seq [42]. This method has been used to show that an increase in transcriptionally silent proviruses correlates to an increased frequency of integration in non-genic DNA. Recently, integration site analysis was performed on several tissues from PWH, showing that HIV-1 integrates more in non-genic, accessible DNA in the brain than in other tissues along the gastrointestinal tract and in cells in the blood [194].

##### Limitations

All methods for integration site analysis rely on PCR-based amplification of the target, which creates limitations for the integration site analysis. PCRs are less efficient in amplifying longer amplicons, and therefore, PCR-based amplification leads to a bias towards shorter amplicons [195]. In addition, incomplete elongation can lead to PCR recombination [196]. Another limitation of integration site analysis methods is that cross-contamination during preparation for sequencing and carry-over from sequential run compromises the reliability of the results [196]. Finally, mispriming and errors in amplification can both lead to misinterpretation of integration site analysis [196]. Wells et al. developed a specific pipeline to process integration site analysis data that addresses these limitations [196].

### 2.3. RNA Assays

#### 2.3.1. HIV-1 RNA Species as Biomarker

Measuring vRNA levels provides another approach to quantify, characterize, and study the HIV-1 reservoir. Integrated proviruses can produce a range of distinct RNA species. In addition to usRNA, msRNA, and other RNA splice variants, TAR RNA—a hairpin structure formed within the first 59 nucleotides of viral transcripts—and readthrough chimeric vRNAs are also transcribed in infected cells [197,198]. When designing a study in which the transcriptionally competent HIV-1 reservoir is measured, this wide diversity of transcripts has to be considered

Additionally, it is crucial to functionally distinguish between vRNA species targeted in the assay in terms of their distinct predictiveness and/or contribution to viral replication and immune activation. Commonly quantified vRNA species in reservoir RNA assays are either cell-associated usRNA or msRNA (Table 1). The detection of *tat*/*rev* msRNA has been proposed as a proper surrogate for virus production, as defective proviruses often encompass deletions in the *tat* and *rev* genes, and Tat translation is essential for productive infection [50,199]. Although usRNA serves as the viral genome and the coding transcript for Gag-Pol polyproteins, it is found to be less predictive of viral replication than msRNA, as usRNA marks the early phase of the viral life cycle [200]. In contrast, usRNA functions as a predictive marker for immune activation as it can be sensed by intracellular innate immune pathways of infected macrophages, which leads to interferon response and pro-inflammatory cytokine release and subsequent T cell dysfunctionality [201]. The immune activation associated with usRNA is observed in both blood and tissue compartments [202,203]. In the lymph nodes, usRNA is shown to correlate with increased activation of CD8+ T cells [203]. Importantly, as described, most integrated proviruses are 3′ mutated or deleted, rendering these therefore unable to generate intact msRNA [67]. Interestingly, defective proviruses have been shown to produce novel usRNA species that retain open reading frames for *gag* and *pol* [204]. Given the distinct predictive value of msRNA for viral replication and the predictive value of usRNA for immune activation and the active reservoir, vRNA cannot be measured as being one molecular intermediate with one interpretation. The primer combinations used to target either msRNA or usRNA must therefore be selected carefully, with the biological indication of the RNA species in mind.

#### 2.3.2. The Inducible Versus the Active Reservoir

The “transcriptionally inducible reservoir” is a term that is often used to refer to the subpopulation of reservoir cells that produce msRNA after a single round of reactivation in culture assays. This subpopulation of reservoir cells that can produce msRNA is clinically relevant to study, as these cells are more likely to contribute to viral rebound during ART interruption [200]. However, it is important to note that partially transcribed genomes might be detected in this assay, depending on the type of RNA targeted in the inducible RNA assay.

In contrast to the inducible reservoir, the active reservoir contains transcriptionally leaky CD4+ T cells that do not require stimulation to measure vRNA, as they generate baseline levels of usRNA transcripts [40,41,52]. This transcriptionally active HIV-1 reservoir is highly relevant to measure because of its potential implications in chronic immune activation [217]. This spectrum of transcriptional activity in the HIV-1 reservoir (from deep latency to transcriptionally active) is therefore crucial to consider during assay selection when aiming to use vRNA as a proxy for replication-competent proviruses.

Assays designed to assess the HIV-1 reservoir on the vRNA level can broadly be divided into three categories: (1) inducible RNA assays, (2) NGS approaches, and (3) in situ hybridization techniques. Each of these methods measures distinct aspects of HIV-1 transcriptional activity and is therefore suited to address different biological questions (Table 1). It is also important to recognize that most assays are restricted to detecting either a specific subset or a single species of viral transcripts, despite the broad diversity of vRNAs present within infected cells.

#### 2.3.3. (Inducible) RNA Assays

The transcriptionally inducible reservoir is commonly measured using culture assays where isolated CD4+ T cells are potently activated (e.g., by anti-CD28/anti-CD3, PMA and ionomycin or LRAs), followed by a single round or semi-nested ddPCR or RT-qPCR to quantify either the intracellular vRNA (cell-associated, CA vRNA) or the vRNA in the supernatant (cell-free, CF vRNA). Both readouts of CA and CF vRNA address different biological questions, and both have notable limitations.

RNA levels are commonly quantified using reverse transcription qPCR (RT-qPCR), which converts vRNA into dsDNA for detection through amplification. The primers used for the RT-qPCR determine the distinct type of vRNA species that is measured and therefore require careful consideration. PCR methods have been developed for usRNA [122,136,218], msRNA [153,218], TAR RNA [219], and chimeric RNA [122,197]. After activating CD4+ T cells, cells can either be analyzed in bulk or in a limiting dilution format, which again diverges which biological questions can be assessed. Bulk assays do not quantify the inducible reservoir but allow for fast quantitation of, for instance, LRA potency in dose–response experiments [122,220,221,222]. Limiting dilution assays, on the other hand, allow for an estimation of reservoir size as number of inducible cells per million CD4+ T cells [123,136,153,154,222]. For a comprehensive overview of the literature on bulk and limiting dilution assays to assess the inducible reservoir of HIV-1 subtype B, we refer the reader to a review by Plantin et al. [223].

In 2015, Procopio et al. introduced the *tat*/*rev* induced limiting dilution assay (TILDA) where CD4+ T cells are activated with PMA and ionomycin, plated in limiting dilution, and prepared for cDNA synthesis [153]. In TILDA, *tat*/*rev* msRNA transcripts are detected using a sensitive qPCR [153,224]. By using a limiting dilution method, the maximum likelihood of the number of reservoir cells per million CD4+ T cells can be approximated. TILDA has become the golden standard assay to assess the transcriptionally inducible reservoir because of its robustness, interlaboratory reproducibility, and technically straight-forward methodology to implement [18,225]. Since this widespread implementation of TILDA in research and clinical trials, researchers have aimed to improve the sensitivity of the assay. Pezzi et al. enhanced the lower limit of detection by using isolated RNA as input instead of whole cells, keeping most of the protocol the same [226]. Similarly, Massanella et al. developed a limiting dilution assay where after 72 h of anti-CD28/anti-CD3 reactivation, CA vRNA levels were measured for *gag* usRNA and *tat*/*rev* msRNA [123]. Regardless of the increased sensitivity of these assays, the original TILDA has advantages, as it requires less labor and lower cell input numbers.

Both bulk RT-qPCR and TILDA utilize single-round or semi-nested RT-qPCR to quantify vRNA. In semi-nested RT-qPCR protocols, cDNA synthesis is followed by manual handling of the pre-amplified product, which increases the labor of this technique and the risk of cross-contamination. Recently, the specific quantification of inducible HIV−1 by RT-LAMP (SQuHIVLa) assay has been developed as an alternative tool to quantify the inducible reservoir [154]. Reverse transcription loop isothermal amplification (RT-LAMP) uses two polymerase enzymes to achieve reverse transcription and amplification of the target amplicon in a single reaction [227]. For RT-LAMP, the formation of a dumbbell-shaped DNA structure is critical for the exponential amplification of the target sequence [227]. SQuHIVLa provides greater specificity than TILDA by using 6–8 primer binding sites that span the *tat*–*rev* region, whereas TILDA uses a total of 4 primers. The design of the SQuHIVLa primers positions the intron between *tat* and *rev* genes within the loop region of the dumbbell structure, causing the loop to be unstable, which prevents aspecific amplification. Consequently, SQuHIVLa specifically targets msRNA over proviral DNA or usRNA. SQuHIVLa can be used as a more sensitive, less expensive, and time-saving alternative to semi-nested RT-qPCR methods such as TILDA [154].

Important to note is that Martin et al. developed an assay to assess the intactness of the RNA present in unstimulated cells called intact viral RNA assay (IVRA) [210]. This assay utilizes RT-ddPCR to amplify and detect RNA regions *psi* and RRE, similarly to the IPDA. This reservoir quantitation tool provides a very interesting approach to assess the baseline active reservoir and its intactness but could also be adapted to be used as an insightful inducible reservoir assay [210].

##### Limitations

A key limitation to the inducible RNA assays is that, regardless of its predictive value, not all reservoir cells produce infectious virus after induced *tat*/*rev* msRNA expression. Post-transcriptional or (post-)translational blocks can still interfere with virion formation, and therefore both assays present an overestimation of the actual inducible reservoir size [59,228]. A limitation for TILDA concerns the risk of proviral DNA amplification, which would contribute further to the overestimation of the inducible reservoir [154]. Another crucial limitation of inducible RNA assays is that the single round of T cell activation might not be sufficient to reactivate all reservoir cells that would rebound after ART cessation. Ex vivo stimulation to evoke *tat*/*rev* msRNA expression is artificial, and this would point towards a possible underestimation of the reservoir.

#### 2.3.4. Next Generation Sequencing-Based vRNA Assays

The caveats associated with PCR-based assays, such as HIV-1 sequence diversity, the chance of detecting false positives, and the overestimation of the reservoir, can be overcome by emerging NGS methods. The various single-cell RNA (scRNA) sequencing methods and their applications for HIV-1 research have been described in depth elsewhere [179,229]. For example, scRNA sequencing enables the simultaneous characterization of viral and host transcripts, allowing for detailed profiling of the transcriptome of HIV-positive CD4+ T reservoir cells. Using sequencing of CA vRNA and HIV-1 DNA (CARD-SGS), the fraction of cells with integrated HIV-1 proviruses that portray transcriptional activity can be determined [41]. Additionally, Clack et al. developed FIND-Seq to define the expression patterns of HIV-1 DNA+ memory CD4+ T cells by sorting HIV-1 DNA negative and positive cells after droplet-based single-cell *gag* amplification [230].

Aside from characterization of the transcriptome of reservoir cells, NGS methods have also been implemented to quantify the inducible reservoir. For example, the EDITS assay (envelope detection by induced transcription-based sequencing) is an NGS assay that leverages PMA and ionomycin stimulation, RNA isolation, and RT-PCR amplification of *env*, followed by sample-specific barcoding to quantify the inducible viral reservoir [211]. The EDITS was compared to the TILDA and used to reveal that the inducible reservoir is significantly lower in women compared to men, highlighting the importance of considering sex-specific differences in any reservoir quantitation study [211].

Another sequencing approach, HIV SortSeq, enables simultaneous quantitation of the inducible reservoir and the characterization of the transcriptional profile of HIV-positive cells [70]. In this method, CD4+ T cells are stimulated with PMA and ionomycin followed by fixation, permeabilization, and hybridization, with HIV-1-specific probes sets targeting both the 3′ and 5′ ends of the viral genome. The cells are subsequently sorted for downstream single-cell transcriptomic analysis. To address HIV-1 sequence diversity, a total of 96 probes against highly conserved regions of the provirus were used during hybridization. Validation of HIV SortSeq in samples of non-B subtypes would inform how applicable the HIV SortSeq approach is across diverse viral subtypes [70].

##### Limitations

A key limitation of NGS-based methods is that the data analysis of NGS-based reservoir quantitation is more time-intensive and laborious than that of limiting dilution assays like TILDA and SQuHIVLa. However, assays like SortSeq have the great advantage of combining reservoir quantitation with transcriptomic information about the reservoir cells, which is impossible for qPCR plate-based TILDA and SQuHIVLa.

#### 2.3.5. In Situ Hybridization Techniques

In situ hybridization (ISH) refers to the technique where a modified nucleic acid strand (or probe) complementary to the RNA or DNA of interest is used to localize the RNA or DNA sequence in a section of tissue (in situ). In the early studies that implemented ISH techniques in HIV-1 research, vRNA was visualized in lymph nodes [231]. Next-generation ISH techniques, including FISH-Flow and RNAscope, allow for the detection of vRNA+ cells using flow cytometry or microscopy, respectively. FISH-flow is a powerful assay as it detects *gag*-*pol* mRNA and p24 protein on a single-cell level using conventional flow cytometry [156,212,213,214]. This combination of simultaneous vRNA and protein detection on single cells reduces the chance of false positive events and improves specificity of detection. FISH-Flow therefore quantifies a subset of reservoir cells that are translationally competent. FISH-Flow also has the potential to target cellular factors in a multiplexed manner, such as rare cytokines and host cell activation markers, simultaneously with vRNA and protein [112,232]. Where FISH-Flow is used to stain and analyze PBMCs from blood, RNAscope enables the analysis of the HIV-1 reservoir within various tissue compartments with high sensitivity [155,233]. RNAscope has proven valuable in the characterization of anatomic compartments that harbor reservoir cells. With this method, probes specific for vRNA (and/or HIV-1 DNA) are added to formalin-fixed paraffin-embedded (FFPE) tissue slides [155,234]. The signal of those probes is then amplified and detected by confocal microscopic analysis. The combination of RNAscope with DNAscope is very valuable in the analysis of which tissue compartments are infected, and which are also transcriptionally active [234].

##### Limitations

A key limitation of the RNAscope assay is that the data is semi-quantitative, since signal intensity does not always correlate linearly with viral transcripts per cell [235]. For the FISH-Flow protocol, a major limitation is that the method is expensive, laborious, and requires high cell input (10–20 million CD4+ T cells), which limits its use in clinical settings [236]. For both ISH techniques, autofluorescence and non-specific probe binding can cause false positives, which affects assay reliability [237].

### 2.4. Protein Assays

The HIV-1 genome gives rise to fifteen viral proteins that are subdivided into structural, regulatory, and accessory proteins [28]. Infected cells generate high numbers of Gag polyprotein, which is why HIV-1 protein assays primarily measure the highly abundant p24, the main capsid protein encoded on the *gag* gene. Studying the translationally competent reservoir is clinically relevant, not only because viral protein presence is a proxy for virion production, but also because HIV-1 proteins are potent immune antigens [238]. Beyond triggering the immune system, HIV-1 proteins are often the target for immunomodulatory cure strategies, and therefore also require tools to accurately measure HIV-1 protein. For reservoir quantitation purposes, measuring the translationally competent reservoir filters out the reservoir cells that are transcriptionally active but do not produce the correct transcripts for viral protein production. It is, however, important to realize that defective proviruses have been reported to still give rise to viral proteins [57]. These alternative peptides have been shown to activate the immune system and may be another underlying cause of the low-level inflammation and associated health issues observed in PWH (reviewed by Kuniholm et al. [239]). These proteins not only induce immune responses but can also interfere with protein as a proxy measurement for replication-competent viruses [57,204].

#### 2.4.1. p24 Assays

The gold standard for p24 viral protein detection has traditionally been the p24 enzyme-linked immunosorbent assay (ELISA) [240,241]. However, the sensitivity of traditional ELISAs fails to detect levels of p24 that can be observed in reactivation and reservoir studies. With the development of the digital single-molecule assay (SIMOA), the ultra-sensitive detection of p24 protein levels was made possible at a 1000-fold lower limit of detection than in traditional ELISAs [242]. In a digital SIMOA, target proteins are captured on antibody-coated magnetic beads, which are then individually isolated in femtoliter-sized wells and treated with a sandwich antibody containing enzymatic substrate. Fluorescent signal after enzymatic treatment is subsequently measured with a high-resolution camera to detect the positive wells, which is translated to the exact concentration of target proteins in a sample. Because of its extreme sensitivity and therefore minimal sample input requirements, the SIMOA following reactivation (induced p24 SIMOA) has been recommended for clinical trial readouts for PBMCs and tissue samples [18]. The p24 SIMOA can be used to directly measure induced or baseline protein expression in a bulk sample of PBMCs or tissue. Alternatively, SIMOA can also be coupled to a viral outgrowth assay, also referred to as the digital ELISA viral outgrowth or DEVO assay [132].

P24 quantitation can also be achieved using the FISH-Flow assay described earlier, where single cell vRNA detection is combined with p24 protein detection using flow cytometry. Another viral protein assay that was validated to measure p24 at the single-cell level is called the viral protein spot (VIP-spot) assay [216]. The set up of the VIP-spot assay is similar to the traditional enzyme-linked immunospot assay (ELIspot) [243] and utilizes anti-CD3 and anti-CD28 antibodies to reactivate the CD4+ T cells, combined with anti-p24 antibodies to capture the HIV-1 p24 antigen. The authors validated the assay by showing a strong correlation between VIP-Spot values and total and intact HIV-1 DNA levels.

##### Limitations

The main limitation associated with measuring protein as a proxy for viral replication is that the majority of the reservoir harbors defective proviruses, which are known to produce abortive peptides [57]. This might lead to an overestimation of the replication-competent reservoir. Protein assays that require ex vivo stimulation, like the induced p24 SIMOA or VIP-Spot, might underestimate the replication-competent reservoir because of the dependency on induction efficiency [50,136]. Downstream of the protein measurement in the VIP-Spot assay, the reactivated cells cannot be used in further flowcytometric analysis, which represents one of the major limitations of this assay. Finally, it should be noted that p24 is commonly targeted for protein detection due to its high abundance. However, as HIV-1 encodes fifteen viral proteins, the detection of p24 alone does not necessarily indicate the presence of all components required for the formation of infectious virions.

### 2.5. Studying the Reservoir of Non-B HIV-1 Subtypes

**Figure 3:**
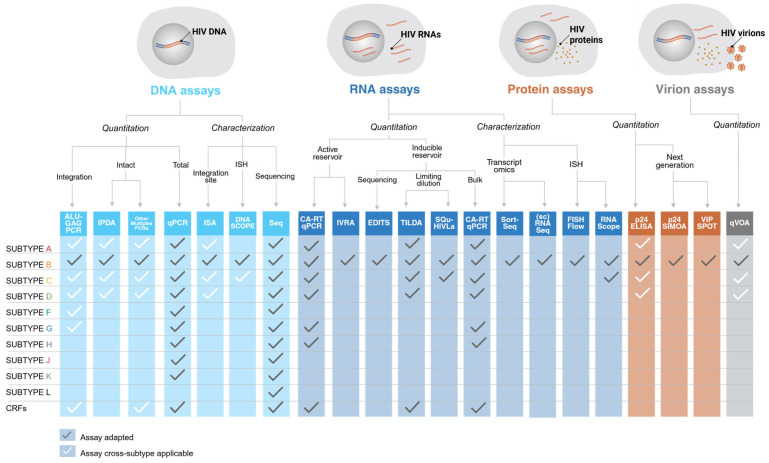
**Overview of reservoir quantitation tools adapted to B and non-B HIV-1 subtypes within group M.** Reservoir quantitation tools are categorized according to the molecular target of the HIV-1 life cycle measured in the assay (DNA, RNA, protein, or virions). Per reservoir quantitation/characterization tool, the applicability of the assay for distinct HIV-1 subtypes is visualized with a check mark. A distinction between newly adapted assays and cross-subtype applicable assays is indicated by colored check marks (see legend). ISH, in situ hybridization; qPCR, quantitative PCR; Seq, sequencing. Created in BioRender. Tiggeler, A. (2026) https://BioRender.com/63a3ciq.

Many of the technologies that have been developed to characterize the HIV-1 subtype B reservoir have also been used or adapted to study the non-B HIV-1 reservoir (Figure 3). In the following section, we discuss the methods that have already been adapted to non-B HIV-1 subtypes, the findings that have been made when these techniques were applied to clinical samples of people with non-B HIV-1 subtypes, and limitations that remain to adapt all techniques for non-B reservoir characterization and quantitation.

#### 2.5.1. Reservoir Characterization and Quantitation Tools Adapted for Non-B Subtypes

##### Viral Outgrowth Assays

The golden standard for quantitation of the replication-competent reservoir, the qVOA, has been used in cohort studies of people with non-B HIV-1 subtypes on two occasions. Prodger et al. performed qVOAs with a p24 ELISA on samples from people with HIV-1 subtype A, D, or an A/D recombinant [244]. The authors observed a smaller reservoir in people with HIV-1 subtype A, D, or an A/D recombinant than in people with HIV-1 subtype B. Similarly, Abrahams et al. used the qVOA on samples from people with HIV-1 subtype C [245]. People with HIV-1 subtype C were shown to have a smaller reservoir if cART was initiated during acute infection rather than chronic infection. This finding substantiated previous reports of the effects of early cART initiation on reservoir size [246].

qVOAs with RNA as a read-out have to date not been performed on samples from people with non-B HIV-1 subtypes.

##### DNA Assays

For the different DNA assays described in this review, many assays have already been adapted to non-B subtypes (Figure 3). For all subtypes, multiple isolates have been sequenced, and for the majority of the HIV-1 subtypes, PCR primers have been designed and validated for quantitation of total HIV-1 DNA levels. The adaptations of more complex assays such as the multiplexed PCR assays, integrated DNA assays, and the integration site analysis assays, will be discussed here in more detail.

##### Intact Proviral DNA Assays

The IPDA as described by Bruner et al. is commonly used for reservoir quantitation and has been recommended for use in clinical studies [18]. Since clinical studies increasingly include people with non-B subtypes, a chip-based adaptation of the IPDA has been tested on clinical samples with subtypes B, CRF01_AE, CRF02_AG, A, C, F, G, and others [162]. The assay showed highly variable detection of the subtypes, showing that the IPDA is unsuitable for detecting intact provirus of subtypes CRF01_AE, C, and F. One resolution is to adapt the method to different subtypes. Currently, an IPDA for subtypes B and C and an IPDA for subtypes A1 and D are available [145,146]. These assays have, however, been validated in limited clinical samples. The IPDA-B/C showed strong correlation to the FLIPS assay (r = 0.92) but has only been validated in five subtype C samples [146]. In addition, Reddy Arikatla et al. have shown through in silico analysis that this IPDA is mostly adapted to subtype C sequences from South Africa and has increased mispriming with subtype C sequences from India [247]. The increased mispriming could lead to decreased sensitivity in assays developed on geographically biased sequences. Similarly, it must be noted that while the IPDA-A1/D showed strong correlation to the original IPDA when assessing subtype B samples (Spearman’s ρ = 0.96), the IPDA-A1/D was developed for 32 sequences from 23 individuals from the Rakai region in Uganda [145]. These assays should be validated on more clinical samples to assess the effects of potential inter-subtype polymorphisms.

The IPDA-5T was also adapted to quantify reservoirs of multiple subtypes, the cross-subtype (CS) IPDA [151]. Degenerate base pairs were introduced to the original primers to match sequences of subtype A, B, C, D, and CRF01_AE. The CS-IPDA was validated with at least five samples from each subtype and was able to more efficiently detect intact proviral DNA in non-B HIV-1 subtypes.

Finally, a more thorough approach to adapt assays to non-B subtypes is to adjust the primer and probe sequences to donor- or cohort-specific sequences. Gunst et al. adapted the primers of the IPDA to each donor sample [108].

##### Integrated DNA Assays

Another approach to adapt primer-based assays to multiple HIV-1 subtypes was demonstrated by the adaptation of the *Alu*-LTR PCR. Vandergeeten et al. developed a cross-subtype *Alu*-LTR PCR and showed that the assay could successfully amplify the HIV-1 LTR of subtypes A, B, C, D, and CRF01_AE [205]. Interestingly, they found that integrated DNA levels varied strongly between subtypes. Isolates from subtypes A and C had particularly lower levels of integrated HIV-1 DNA than an isolate from subtype B, while an isolate from subtype D had a much higher level. The method was only validated on one isolate from each subtype, but this technique was later applied to a large HIV-1 cohort [206]. Integrated HIV-1 DNA was detected in 59% of the samples (n = 312). At least five samples of subtypes A, B, C, D, F, G, and multiple recombinants were used to assess subtype-specific differences. This assessment showed that integrated HIV-1 DNA levels were lower in subtype C (n = 25) and CRF02_AG (n = 31) and higher in subtype D (n = 12) compared to subtype B (n = 66), which was in line with previous findings.

##### Integration Site Analysis Assays

Kohio et al. developed an integration site analysis that can be performed on subtypes A, C, and D. A meta-analysis of integration site analyses for subtypes A, B, C, and D using partly published data and new experimental data suggested that there are subtype-specific preferences for integration sites [248]. The authors showed that subtype C was integrated in more transcriptionally silent regions than subtypes A, B, and D.

##### RNA Assays

Several methods to measure the inducible reservoir on the RNA level have been adapted for non-B subtypes (Figure 3). Since all inducible RNA assays measure vRNA using either RT-qPCR or RT-LAMP amplification, the adaptation to different subtypes mainly requires the adaptation of primers and probes.

##### (Inducible) RNA Assays

One assay that has been adapted for non-B subtypes is TILDA, which has been adapted for subtypes A, C, D, and E [89,207,208,209]. The first adaptation of TILDA was made for HIV-1 subtype C [89]. C-TILDA relies on the exact same principle as the TILDA for subtype B, except the primers and probe were designed for conserved regions of *tat*-*rev* exons of subtype C [89]. In this study, the researchers showed that B-TILDA failed to detect inducible reservoir cells in subtype C samples, clearly indicating the importance of subtype adaptation [89]. In two other studies, primers and probes were designed to detect msRNA of subtypes CRF01_A/E, A, and CRF02_A/G [207,249]. Alternatively, Mishra et al. opted to design one primer set that can universally detect HIV-1 msRNA from subtypes A, B, C, D, and E, and termed this U-TILDA (universal TILDA) [209]. This was achieved by aligning a range of subtype sequences using the LANL sequence database and designing primer and probe sequences complementary to exon 1 (non-coding) and exon 7 (*rev*-coding exon), instead of exon 5 (*tat* coding exon) and exon 7 (*rev*-coding exon). Both exons 1 and 7 are present in all viral transcripts regardless of splicing differences. However, the length of the amplicon of usRNA was proven to be refractory to amplification given the larger size [208,209]. U-TILDA clearly holds promise for clinical implementation to detect an enhanced breadth of HIV-1 subtypes, but since its publication in 2022, it is yet to be applied in new, published studies.

Another example of accommodating subtype adaptation for reservoir quantitation tools is the publication of SQuHIVLa, which was accompanied with a guide to design primers for non-B subtypes. The paper includes primer and probe sets for subtypes B and C, and the assays were validated and tested on PBMCs from people with HIV-1 subtypes B or C [154].

Additionally, Joussef-Piña et al. modified the original EDITS assay to measure inducible CA HIV-1 *env* mRNA in both B and non-B HIV-1 subtypes. The researchers developed primer sets that bind to highly conserved regions in the HIV-1 genome and thoroughly validated these using ex vivo infection experiments for 32 diverse HIV-1 group M strains. This study revealed that the peripheral HIV-1 reservoir of the non-B Ugandan cohort (31% A1, 64% D, or 5% C) was 4-fold smaller and 2-fold more genetically diverse than the US subtype B cohort.

##### In Situ Hybridization Techniques

Lastly, the reservoir ISH characterization technique RNAscope has been adapted to study the non-B HIV-1 reservoir. Deleage et al. generated and validated RNAscope probes for HIV-1 subtypes A, CRF-AE, C, and D [155]. Interestingly, using these probes for subtype C, the brain has emerged as a much smaller tissue reservoir than reported in people with HIV-1 subtype B, indicating the importance of HIV-1 subtype-specific research [88]. Accordingly, subtype-specific RNAscope has been used to characterize several different HIV-1 subtype C tissue reservoirs [250,251]. In contrast to subtype-specific RNAscope, one single set of probes has been validated and used to detect *gag*-*pol* RNA from both subtype B and dominant strains in China CRFO7_BC and CRF01_AE [252]. Likewise, Zhang et al. showed that RNAscope HIV-1B probes were applicable to detect HIV-1 vRNA following LRA activation in subtypes A1, B, C, and CRF01-AE [233].

##### Protein Assays

The subtype agnostic applicability of p24 assays has been extensively explored in HIV-1 diagnostics but has not been thoroughly studied for the purpose of measuring the translationally competent HIV-1 reservoir. In 2003, Ribas et al. presented a modified p24 ELISA for p24 antigen detection in plasma, where the p24 protein is heat-denatured prior to detection, which allows for the detection of various subtype recombinant forms of p24 [253]. These authors set a great example, where studying the intra-subtype sensitivity in the assay is one of the main objectives of the study. A study comparing diagnostic HIV-1 antigen/antibody (Ag/Ab) tests (simultaneously measuring p24 levels and antibodies against HIV-1) revealed variable sensitivity in detecting HIV-1 non-B and non-M strains [254]. Later, another evaluation of FDA-approved HIV Ag/Ab tests demonstrated variable clinical specificity across HIV-1 subtypes [255]. Clearly, in the context of early diagnostics, p24 detection tools have been closely evaluated for their applicability for non-B subtypes. With the continuous advances of p24 antigen immunoassays, fourth-generation antigen/antibody (Ag/Ab) tests are now in clinical use; however, ongoing evaluation against current circulating HIV-1 subtypes will remain an important issue [256,257].

In contrast, the validation of reservoir quantitation tools that measure viral protein such as p24 SIMOA and VIP-SPOT remain mostly undescribed for non-B subtypes. For instance, the novel VIP-SPOT assay has recently been validated to assess inducible reservoir dynamics in a cohort predominantly consisting of subtype B [216,258]. As the technique has proven effective for subtype B, it holds significant promise for validation across additional subtypes in future studies. Similarly, for p24 SIMOA, the breath of antibody reactivity between subtypes remains mostly elusive, and the published literature is heavily dominated by a focus on HIV-1 subtype B.

#### 2.5.2. Limitations and Perspectives to Adapt Reservoir Characterization and Quantitation Tools for Non-B Subtypes

In this section, we discuss factors that limit the use of reservoir quantitation and characterization techniques developed for subtype B to non-B subtypes. All of these limitations arise from genetic variation observed between subtypes; however, it is important to note that genetic variation also exists within the quasispecies per subtype. Here, we focus on subtype-specific genetic variations that may prevent the application of a method developed for subtype B to non-B HIV-1 subtypes.

##### Nucleotide-Based Assays

Many of the assays discussed in this review utilize primers and probes for the amplification and detection of HIV-1-specific nucleotide sequences. These assays are crucial for quantifying DNA and RNA intermediates. Primers and probes used in these assays need to be complementary to the target sequence to bind, and mismatches form a major limitation to applying established techniques to targets that have poorly conserved target sequences. While these genetic variations occur naturally between sequences within subtypes, the genetic variation between subtypes may be so large that mismatches occur systematically. Levy et al. previously showed that primers may still be able to amplify sequences if less than six mismatches exist between the primer and target sequences [150]. However, mismatches may lead to reduced amplification and detection that may be subtype-specific. To be able to compare results of nucleotide-based assays between subtypes, cross-subtype, subtype-specific, cohort-specific, or donor-specific primers can be designed and validated. Alternatively, an approach using many different primers and probes targeting a larger amplicon can be used to account for genetic variation within and between subtypes.

Ideally, any reservoir quantitation technique can be used on every sample. When it comes to nucleotide-based assays, it would therefore be preferable to have cross-subtype primers and probes that have a similar efficiency for every subtype. This approach was used to adapt the *Alu*-LTR PCR to non-B subtypes, and this cross-subtype *Alu*-LTR assay enables quantitation of integrated DNA regardless of subtype [205]. Cross-subtype primers and probes can only be designed for highly conserved regions within the HIV-1 genome. Not every nucleotide-based assay targets a highly conserved region, however. Consequently, this approach will not be useful for the adaptation of all assays to non-B subtypes.

When cross-subtype primers and probes cannot be designed, or if they are not comparatively efficient between subtypes, one option is to design subtype-specific primers and probes. Already, several assays, such as the IPDA [146], TILDA [153], and the SQuHIVLa [154], have been adapted to be subtype-specific using consensus sequences based on sequences published in the Los Alamos National Laboratory HIV-1 sequence database. It is crucial that these newly adapted primers and probes be validated on a global variety of samples to ensure similar efficiency within the subtype. In absence of global validation, there is a risk of a geographical bias, as was shown by Reddy Arikatla et al. [247].

Thirdly, primers and probes can be adjusted to be donor-specific or cohort-specific—an approach Gunst et al. used to be able to perform IPDAs on samples with different HIV-1 subtypes [108]. However, this approach introduces other variables that may influence reproducibility, such as differences in efficiency. Donor-specific nucleotide-based assays are therefore not suitable for reservoir size comparison between donors and should only be used for paired comparisons in longitudinal studies. In addition, these assays require sequencing, which adds to the labor, duration, and cost of the assay, making them unsuitable for high-throughput data analysis.

Lastly, multiple in situ techniques rely on a collection of probes that target a large amplicon. With this approach, polymorphisms between samples are less likely to result in low sensitivity, or even assay failure, because there are many possible binding sites. This approach is applied in the FISH-Flow protocol that utilizes a set of 50 probes spanning over the *gag*-*pol* region to detect vRNA (Figure 2) [156]. Therefore, hybridization and signal amplification are very specific, and there is a high tolerance for polymorphisms, which might allow for application of the method to non-B HIV-1 subtypes. While this technique is less applicable to primer-based amplification reactions, there are multiple multiplexed DNA assays that target multiple amplicons to reduce the risk of assay failure due to polymorphisms, such as the Q4PCR and the Rainbow assay [148,152].

In the development of nucleotide-based assays to quantify and characterize the HIV-1 reservoir, the continuous genetic evolution of HIV-1 must be carefully considered. Assay compatibility must therefore be continuously optimized for all subtypes, including B and non-B variants. Validation of nucleotide-based assays in clinical samples worldwide is essential to be able to compare HIV-1 reservoir sizes reported between subtypes, globally and in time.

##### Protein-Based Assays

When using p24 as a subtype agnostic read-out for the translationally competent reservoir, it is important to consider the intra-subtype differences in the amino acid sequence of p24. Failure to detect HIV-1 antigens from non-B subtypes using p24 ELISAs has been described as early as 1998, suggesting epitope differences in p24 for antibodies used in this assay [259]. More recently, bioinformatic analysis of p24 amino acid diversity among HIV-1 M, O, P, and N groups, revealed that, indeed, the secondary structure of p24 varied across subtypes [260]. Regardless of structural differences in p24, all HIV-1 variants showed over 80% sequence conservation compared to HXB2, the subtype B reference sequence. Single amino acid variations in the targeted epitopes of p24 can lead to failure of detection and false negative results or underestimation of p24 presence after qVOAs or direct protein measurements [261]. The use of broadly reactive antibodies targeting conserved p24 epitopes is therefore crucial in p24 assays to detect all HIV-1 non-B isolates.

##### Inducible Assays

Stimulation-based assays such as qVOA or inducible RNA assays rely on potent immune activators to induce viral transcription (and translation) in latently infected cells. However, the transcriptional and translational response to stimulation may differ extensively between subtypes, due to genetic variation in the 5′ LTR sequences, resulting in distinct transcription factor binding sites, chromatin regulation, and the associated response to signaling pathways activated by stimuli. This subtype-specific response to activating stimuli could complicate the interpretation of the read-outs. It may, for instance, contribute to the observation of a smaller reservoir size in people with HIV-1 subtypes A, C, and D, as determined with qVOA [244,245].

## 3. Discussion

Here, we have discussed the methods that have been developed for characterization and quantitation of the distinct molecular compartments of the HIV-1 reservoir. While the golden standard for quantifying replication-competent virus remains the qVOA, a plethora of methods have been developed that measure different molecular intermediates of the HIV-1 infection cycle as a proxy eliminating the labor, time and cost associated with the qVOA Quantitation of these intermediates can be used to answer different pre-clinical and clinical research questions.

The focus of HIV-1 cure research has been on HIV-1 subtype B, which has led to an underrepresentation of other HIV-1 subtypes that make up the vast majority of the global population of PWH. To facilitate a globally effective cure approach, it is crucial that a common guideline of standardized assays with minimal inter-lab variability is established to gain reliable insight into the establishment and longitudinal decay dynamics of the reservoir and its response to therapeutic and curative interventions. This enhanced understanding of subtype-specific reservoir dynamics can guide the selection of appropriate cure strategies. Therefore, standardizing and adapting assays for reservoir quantitation and characterization among subtypes is not only informative, but crucial to clinically address all HIV-1 endemic populations. The choice of standardized tests used on clinical samples should be determined by the biological question, the specificity and sensitivity metrics of the test, the suitability of the tests to all HIV-1 subtypes, and the accessibility of these tests.

As discussed, different molecular intermediates serve to answer different biological questions related to clinical HIV-1 cure trials. For instance, a main interest in HIV-1 cure research is the risk of viral rebound during an analytical treatment interruption (ATI). Previously, Zerbato et al. showed that *tat*/*rev* msRNA levels are indicative of viral rebound after in vivo LRA treatment [200]. Therefore, any cure approach that enters an ATI-containing clinical trial may benefit from assessment of CA msRNA levels at basal or induced states to monitor the potential for rebound in advance of and after an ATI, through methods such as TILDA [153] or SQuHIVLa [154].

Alternatively, molecular intermediates of HIV-1 can potentially serve as biomarkers of chronic inflammation in PWH. Akiyama et al. showed that usRNA can be sensed by innate pathways that drive interferon responses and indirectly induce T cell dysfunction [201]. Similarly, defective HIV-1 DNA can give rise to alternative RNA transcripts and peptides that are known to activate CD8+ T cells [204,239]. Accordingly, measurements of defective HIV-1 DNA with a multiplexed DNA assay and intron-containing usRNA using, for example, a CA usRNA qPCR assay, can be predictive of chronic inflammation. Naturally, the predictive value of these biomarkers has to be validated, which can be accommodated by characterizing cohorts with standardized tests for these measurements. Whether the predictive value of these biomarkers holds true for non-B subtypes remains largely unexplored and should be validated in studies using adapted versions of standardized assays.

Currently, a substantial proportion of reservoir quantitation tests has been adapted to and applied to clinical samples of most prevalent HIV-1 subtypes (Figure 3). However, it is essential that the performance of these assays for different subtypes is properly validated, in particular for nucleotide-based assays. This validation process needs to be continuous to ensure the applicability of the assays as HIV-1 sequences continue to evolve.

Although newly developed assays provide increasingly detailed insights into reservoir biology, many of these novel reservoir quantitation and characterization methods are not suitable for implementation in low- and middle-income countries. Accessibility of reservoir quantitation tools should heavily influence the choice for standardized assays, particularly for low-resource laboratories. Previously, Nakanjako et al. discussed major limitations to pre-clinical and clinical HIV-1 cure research in Africa [262]. Several of these limitations can be addressed in the choice for the quantitation and characterization assays. Many of the techniques described in this review require specific expertise, laboratory equipment, or increased funding due to expensive reagents or intensive labor (Table 1). It is therefore recommended that alternative assays that address these limitations are chosen for the standardization of reservoir quantitation and characterization.

Here, we argue that development of reservoir quantitation and characterization tools for usage in cohort studies and clinical trials should focus on validating adapted and accessible assays so that standardized assays can be chosen for each biological question that is asked in HIV-1 cure research. In clinical research, these validated standardized assays should be used to evaluate the subtype-specific responses to alternative treatments and potential curative treatments. Moreover, pre-clinical HIV-1 research should focus on the characterization of non-B HIV-1 reservoirs. Before cure strategies can be employed globally, these steps are required to understand the reservoir dynamics of all HIV-1 subtypes and how they respond to potential curative treatments.

## Figures and Tables

**Table 1:** **Overview of HIV-1 reservoir quantitation tools.** Reservoir quantitation tools are reviewed for their applicability to different HIV-1 subtypes and their benefits and downsides in high-throughput research. Required infrastructure and estimated price per processed sample is indicated in € signs representing tenfold increases in costs. ddPCR, digital droplet PCR; dPCR, digital PCR; vRNA, viral RNA.

Assay	Target	Read-Out	Subtypes	Benefits	Downsides	Required Laboratory Equipment	Price Indication Per Sample	Ref.
**IPDA**	DNA (intact)	Frequency of CD4+ T cells that contain intact HIV-1 DNA (two regions)	A1, B, C, D	Adapted to multiple subtypes; requires few cells	Overestimation; frequent assay failure due to polymorphisms	ddPCR machine or dPCR machine	€€	[144,145,146,161]
**Q4PCR**	DNA (intact)	Frequency of CD4+ T cells that contain intact HIV-1 DNA (four regions)	B	Reduced overestimation on account (<IPDA); requires few cells	Labor-intensive due to limiting dilution	dPCR machine with four channels	€€	[148]
**Q4ddPCR**	DNA (intact)	Frequency of CD4+ T cells that contain intact HIV-1 DNA (four regions)	B	Less labor-intensive (<Q4PCR); increase sensitivity (>IPDA and Q4PCR); less overestimation (<IPDA and Q4PCR); requires few cells		ddPCR machine	€€	[149]
**IPDA-5T**	DNA (intact)	Frequency of CD4+ T cells that contain intact HIV-1 DNA (five regions)	A, B, C, D, CRF_01AE	Suited to many subtypes; requires few cells	Time-intensive	ddPCR machine	€€	[150,151]
**Triplex Assay**	DNA (intact)	Frequency of CD4+ T cells that contain intact HIV-1 DNA (three regions)	B	Sensitive (>IPDA); requires few cells		ddPCR machine	€€	[147]
**Rainbow Assay**	DNA (intact)	Frequency of CD4+ T cells that contain intact HIV-1 DNA (five regions)	B	Reduced overestimation (<IPDA and Q4PCR); requires few cells		dPCR machine with five channels	€€	[152]
**Integration analysis**	DNA (integrated)	Integrated HIV-1 DNA levels	A, B, C, D, F, G, CRFs	Suited to many subtypes	Overestimation	qPCR machine	€€	[164,165,166,168,169,170,175,176,205,206]
**TILDA**	RNA (msRNA, inducible)	Frequency of msRNA+ CD4+ T cells (after PMA/ionomycin stimulation)	A, B, C, D, E	Adapted to multiple subtypes; high-throughput; requires fewer cells (<qVOA)	Overestimation of the replication-competent reservoir; cross-contamination risk between RT-qPCR and semi-nested amplification	qPCR machine	€€€	[89,153,207,208,209]
**SQuHIVLa**	RNA (msRNA, inducible)	Frequency of msRNA+ CD4+ T cells (after PMA/ionomycin stimulus)	B, C	Reduced cost, less time-intensive, and lower cross-contamination risk (<TILDA), same specificity; adapted to subtype C	Overestimation of the replication-competent reservoir	qPCR machine	€€€	[154]
**IVRA**	RNA (intact)	Frequency of msRNA+ CD4+ T cells	B	Gives important insight in the intactness of the full-length RNA transcripts that are produced		ddPCR machine	€€	[210]
**EDITS**	RNA	Frequency of usRNA+ CD4+ T cells without stimulation	B	Time- and cost-efficient compared to nFL sequencing; high reproducibility	Sequencing-based method requires expertise and infrastructure	PCR machine and ion torrent sequencing system	€€	[211]
**HIV-1 FISH-Flow**	RNA and protein	Frequency of vRNA+ and HIV-1 protein+ cells	B, possibly more (high coverage in probes to conserved regions)	Combined RNA with protein data on single cell level	Labor- and time-intensive, aspecific binding from p24 antibody	Flow cytometer	€€€	[156,212,213,214]
**(induced) SIMOA 24**	Protein (inducible)	Soluble levels of p24 protein in fg/mL	B	Highly sensitive compared to traditional ELISA, low sample input requirement	Requires digital/planar ELISA infrastructure, higher per-sample cost,	Digital immunoassay analyzer	€€€	[215]
**VIP-Spot**	Protein (inducible)	Frequency of p24-producing, translationally competent cells per million CD4+ T cells	B	Detects p24 production as single cell read-out	Labor and time-intensive, not suited for high-throughput experiments	ELISpot reader unit	€€	[216]

## Data Availability

No new data were created or analyzed in this study. Data sharing is not applicable to this article.
